# Complex relationship between serum IGFBP2 and FGF21, dysmetabolism and cognitive function across the clinical spectrum of Alzheimer’s disease

**DOI:** 10.1002/alz.090020

**Published:** 2025-01-09

**Authors:** Caroline Dallaire‐Théroux, Hélèna Denis, Amélie Provencher, Josue Valentin‐Escalera, Manon Leclerc, Cyntia Tremblay, Andréanne Loiselle, Marine Tournissac, Sylvie Belleville, Frédéric Picard, Henrik Zetterberg, Simon Duchesne, CIMA‐Q Group, Frederic Calon

**Affiliations:** ^1^ Université Laval / CHU de Québec, Quebec City, QC Canada; ^2^ Universite Laval, Quebec, QC Canada; ^3^ CHU de Quebec, Quebec, QC Canada; ^4^ Université Laval, Québec, QC Canada; ^5^ Faculty of pharmacy, Université Laval, Quebec, QC Canada; ^6^ Research centre, Institut universitaire de gériatrie de Montréal, Montréal, QC Canada; ^7^ Wisconsin Alzheimer’s Disease Research Center, University of Wisconsin School of Medicine and Public Health, Madison, WI USA; ^8^ Centre de recherche de l’Institut Universitaire de Cardiologie et de Pneumologie de Québec, Quebec, QC Canada; ^9^ Consortium pour l'Identification précoce de la Maladie d'Alzheimer ‐ Québec, Montréal/Québec/Sherbrooke, QC Canada; ^10^ Laval University, Quebec, QC Canada

## Abstract

**Background:**

Chronic conditions such as obesity, dyslipidemia, insulin resistance and metabolic syndrome are found with increased prevalence in older adults and are investigated as risk factors of Alzheimer’s disease (AD). The exact mechanisms by which such conditions may act on the brain to promote AD pathogenesis are still unknown. We aimed to investigate the relationship between peripheral markers of metabolic function, including adiponectin, fibroblast growth factor 21 (FGF21) and insulin‐like growth factor binding protein 2 (IGFBP2), and AD‐associated cognitive decline.

**Method:**

Samples from 288 participants (mean age, 74 [SD, 6] years; 37% males) were obtained through the CIMA‐Q cohort. Participants were classified as cognitively healthy (CH; n=56), subjective cognitive decline (SCD; n=115), mild cognitive impairment (MCI; n=86), and AD (n=31). Demographics, APOE4 status, cognitive testing, anthropometrics, blood and cerebrospinal fluid biochemical parameters and MRI data were compared between clinical groups using one‐way ANOVA and Kruskal‐Wallis tests. Multiple linear regressions were performed to evaluate the association between serum levels of adiponectin, FGF21 and IGFBP2 and clinical, biochemical and neuroimaging data, with adjustment for age and sex.

**Result:**

Higher levels of FGF21 (p=0.01) and IGFBP2 (p<0.001) were found with increasing age. Amongst the three metabolic markers, IGFBP2 was significantly elevated in SCD and AD (p=0.04) compared to CH. Clinical and biochemical evidence of obesity, insulin resistance and dyslipidemia were associated with lower serum adiponectin and IGFBP2, but higher FGF21 (p<0.0001). Significant inverse associations between metabolic markers and clinical scores included FGF21 with Rey Auditory Verbal Learning Test (RAVLT; p=0.002) and IGFBP2 with Montreal Cognitive Assessment (MoCA; p=0.02). Adiponectin levels were not linked to clinical diagnosis nor cognition but were higher in women. Finally, IGFBP2 also correlated with markers of neurodegeneration including higher plasma pTau181 and lower amygdala volume (p<0.05).

**Conclusion:**

While FGF21 and IGFBP2 exhibit opposing trends in metabolically impaired individuals, both markers increase with age and are inversely associated with cognitive performance. However, serum IGFBP2 is more strongly associated with clinical diagnosis and cognitive function than FGF21. These results unveil complex relationships between fluid‐based biomarkers of dysmetabolism and AD, offering new opportunities for diagnosis and treatment.

